# (*E*)-*N*′-(2,5-Dimethoxy­benzyl­idene)-2,4-dihydroxy­benzohydrazide

**DOI:** 10.1107/S160053681001130X

**Published:** 2010-03-31

**Authors:** Jing-Yuan Wei, De-Guang Song, Da-Cheng Wang, Xu-Ming Deng, Ju-Xiong Liu, Bo Liu

**Affiliations:** a College of Animal Science and Veterinary Medicine, Jilin University, Changchun 130062, People’s Republic of China

## Abstract

In the title compound, C_16_H_16_N_2_O_5_, the dihedral angle between the two benzene rings is 4.2 (2)° and an intra­molecular O—H⋯O hydrogen bond generates an *S*(6) ring. In the crystal, mol­ecules are linked into layers lying parallel to the *bc* plane by O—H⋯O and N—H⋯O hydrogen bonds.

## Related literature

For the biological properties of Schiff base compounds, see: Bhandari *et al.* (2008 ▶); Sinha *et al.* (2008 ▶). For Schiff base compounds containing 2,5-dimethoxy­benzaldehyde, see: Wang *et al.* (2009 ▶). For reference structural data, see: Allen *et al.* (1987 ▶).
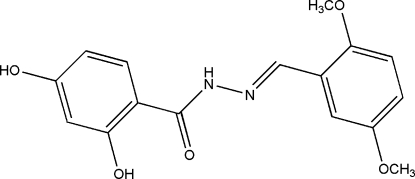


## Experimental

### 

#### Crystal data

C_16_H_16_N_2_O_5_
*M* *_r_* = 316.31
Monoclinic, 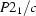

*a* = 7.8600 (16) Å
*b* = 15.358 (3) Å
*c* = 12.425 (3) Å
β = 99.80 (3)°
*V* = 1478.0 (5) Å^3^
*Z* = 4
Mo *K*α radiation
μ = 0.11 mm^−1^
*T* = 295 K
0.18 × 0.17 × 0.15 mm

#### Data collection

Bruker SMART CCD diffractometer
Absorption correction: multi-scan (*SADABS*; Sheldrick, 1996 ▶) *T*_min_ = 0.981, *T*_max_ = 0.984
7757 measured reflections
2626 independent reflections
1698 reflections with *I* > 2σ(*I*)
*R*_int_ = 0.039

#### Refinement

*R*[*F* ^2^ > 2σ(*F* ^2^)] = 0.043
*wR*(*F* ^2^) = 0.112
*S* = 1.02
2626 reflections
212 parameters
H-atom parameters constrained
Δρ_max_ = 0.15 e Å^−3^
Δρ_min_ = −0.20 e Å^−3^

### 

Data collection: *SMART* (Siemens, 1996 ▶); cell refinement: *SAINT* (Siemens, 1996 ▶); data reduction: *SAINT*; program(s) used to solve structure: *SHELXS97* (Sheldrick, 2008 ▶); program(s) used to refine structure: *SHELXL97* (Sheldrick, 2008 ▶); molecular graphics: *SHELXTL* (Sheldrick, 2008 ▶); software used to prepare material for publication: *SHELXTL*.

## Supplementary Material

Crystal structure: contains datablocks global, I. DOI: 10.1107/S160053681001130X/hb5375sup1.cif

Structure factors: contains datablocks I. DOI: 10.1107/S160053681001130X/hb5375Isup2.hkl

Additional supplementary materials:  crystallographic information; 3D view; checkCIF report

## Figures and Tables

**Table 1:** Hydrogen-bond geometry (Å, °)

*D*—H⋯*A*	*D*—H	H⋯*A*	*D*⋯*A*	*D*—H⋯*A*
O1—H1⋯O3	0.82	1.76	2.495 (2)	148
O2—H2⋯O3^i^	0.82	1.92	2.664 (2)	151
N1—H1*A*⋯O1^ii^	0.86	2.17	3.012 (2)	166

Symmetry codes: (i) 
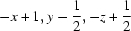
; (ii) 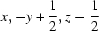.

**Fig. 1.**
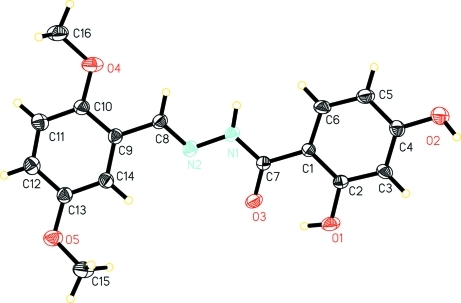
The molecular structure of (I), with displacement ellipsoids drawn at the 30% probability level. The dashed lines indicate hydrogen bonds.

**Fig. 2.**
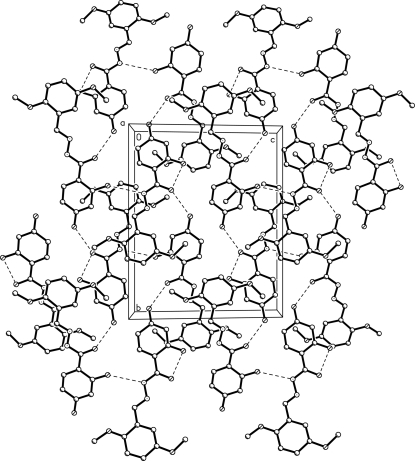
The molecular packing of (I). Intermolecular hydrogen bonds are shown as dashed lines. H atoms not involved in the hydrogen bonds have been omitted for clarity.

## supplementary crystallographic information

### Comment

Schiff base compounds have been of great interest for many years. Some of the complexes derived from Schiff bases have been found to have pharmacological and antitumor properties (Bhandari *et al.*, 2008; Sinha *et al.*, 2008). In this paper, the crystal structure of the title compound, (I), a new Schiff base compound derived from the condensation reaction of 2,4-dihydroxybenzohydrazide with 2,5-dimethoxybenzaldehyde is reported.

The Schiff base molecule of the compound displays a *trans* configuration with respect to the C=N and C—N bonds(Fig. 1). All the bond lengths are within their normal ranges (Allen *et al.*,  1987) and comparable to other Schiff base compounds containing 2,5-dimethoxybenzaldehyde (Wang *et al.*, 2009). The dihedral angle between the two benzene rings is 4.2 (2)°. Intramolecular O—H···O hydrogen bonds are observed(Table 1). Molecules are linked into layers parallel to the *bc* plane by O—H···O and N—H···O hydrogen bonds(Fig. 2).

### Experimental

2,5-dimethoxybenzaldehyde (0.1 mmol, 16.6 mg) and 2,4-dihydroxybenzohydrazide (0.1 mmol, 16.8 mg) were dissolved in a 95% ethanol solution (10 ml). The mixture was stirred at room temperature to give a clear colorless solution. Light yellow blocks of (I) were formed by gradual evaporation of the solvent over a period of three days at room temperature.

### Refinement

All H atoms were placed in geometrically idealized positions, with C—H = 0.93–0.96 Å, O—H = 0.82–0.85 Å and N—H = 0.86 Å. *U*_iso_(H) = 1.2*U*_eq_(C,*N*), and 1.5*U*_eq_(O).

### Crystal data

**Table:**

C_16_H_16_N_2_O_5_	*F*(000) = 664
*M**_r_* = 316.31	*D*_x_ = 1.421 Mg m^−^^3^
Monoclinic, *P*2_1_/*c*	Mo *K*α radiation, λ = 0.71073 Å
Hall symbol: -P 2ybc	Cell parameters from 1224 reflections
*a* = 7.8600 (16) Å	θ = 2.7–22.4°
*b* = 15.358 (3) Å	µ = 0.11 mm^−^^1^
*c* = 12.425 (3) Å	*T* = 295 K
β = 99.80 (3)°	Block, light yellow
*V* = 1478.0 (5) Å^3^	0.18 × 0.17 × 0.15 mm
*Z* = 4	

### Data collection

**Table:**

Bruker SMART CCD diffractometer	2626 independent reflections
Radiation source: fine-focus sealed tube	1698 reflections with *I* > 2σ(*I*)
graphite	*R*_int_ = 0.039
φ and ω scans	θ_max_ = 25.1°, θ_min_ = 2.1°
Absorption correction: multi-scan (*SADABS*; Sheldrick, 1996)	*h* = −9→8
*T*_min_ = 0.981, *T*_max_ = 0.984	*k* = −17→18
7757 measured reflections	*l* = −13→14

### Refinement

**Table:**

Refinement on *F*^2^	Primary atom site location: structure-invariant direct methods
Least-squares matrix: full	Secondary atom site location: difference Fourier map
*R*[*F*^2^ > 2σ(*F*^2^)] = 0.043	Hydrogen site location: inferred from neighbouring sites
*wR*(*F*^2^) = 0.112	H-atom parameters constrained
*S* = 1.02	*w* = 1/[σ^2^(*F*_o_^2^) + (0.0464*P*)^2^ + 0.2321*P*] where *P* = (*F*_o_^2^ + 2*F*_c_^2^)/3
2626 reflections	(Δ/σ)_max_ < 0.001
212 parameters	Δρ_max_ = 0.15 e Å^−^^3^
0 restraints	Δρ_min_ = −0.20 e Å^−^^3^

### Special details

**Table:**

Geometry. All esds (except the esd in the dihedral angle between two l.s. planes) are estimated using the full covariance matrix. The cell esds are taken into account individually in the estimation of esds in distances, angles and torsion angles; correlations between esds in cell parameters are only used when they are defined by crystal symmetry. An approximate (isotropic) treatment of cell esds is used for estimating esds involving l.s. planes.
Refinement. Refinement of *F*^2^ against ALL reflections. The weighted *R*-factor wR and goodness of fit *S* are based on *F*^2^, conventional *R*-factors *R* are based on *F*, with *F* set to zero for negative *F*^2^. The threshold expression of *F*^2^ > σ(*F*^2^) is used only for calculating *R*-factors(gt) etc. and is not relevant to the choice of reflections for refinement. *R*-factors based on *F*^2^ are statistically about twice as large as those based on *F*, and *R*- factors based on ALL data will be even larger.

### Fractional atomic coordinates and isotropic or equivalent isotropic displacement parameters (Å^2^)

**Table:**

	*x*	*y*	*z*	*U*_iso_*/*U*_eq_	
O1	0.5668 (2)	0.18995 (10)	0.34476 (11)	0.0523 (5)	
H1	0.6007	0.2404	0.3438	0.078*	
O2	0.2931 (2)	−0.02763 (10)	0.10807 (13)	0.0623 (5)	
H2	0.3162	−0.0573	0.1635	0.093*	
O3	0.6343 (2)	0.33256 (9)	0.26549 (11)	0.0477 (4)	
O4	0.7730 (2)	0.58885 (10)	−0.13674 (12)	0.0569 (5)	
O5	1.0792 (2)	0.67891 (10)	0.28127 (12)	0.0529 (4)	
N1	0.6247 (2)	0.35121 (10)	0.08522 (13)	0.0390 (5)	
H1A	0.5957	0.3331	0.0192	0.047*	
N2	0.7069 (2)	0.42982 (11)	0.10809 (13)	0.0390 (5)	
C1	0.5091 (3)	0.21858 (13)	0.15178 (15)	0.0329 (5)	
C2	0.5058 (3)	0.16342 (13)	0.24143 (16)	0.0354 (5)	
C3	0.4396 (3)	0.08035 (14)	0.22852 (16)	0.0391 (5)	
H3	0.4436	0.0439	0.2887	0.047*	
C4	0.3674 (3)	0.05154 (14)	0.12604 (17)	0.0421 (6)	
C5	0.3657 (3)	0.10522 (15)	0.03614 (17)	0.0527 (7)	
H5	0.3168	0.0857	−0.0331	0.063*	
C6	0.4356 (3)	0.18663 (14)	0.04918 (16)	0.0451 (6)	
H6	0.4342	0.2218	−0.0119	0.054*	
C7	0.5915 (3)	0.30376 (13)	0.17057 (16)	0.0368 (5)	
C8	0.7456 (3)	0.47279 (13)	0.02779 (17)	0.0386 (5)	
H8	0.7160	0.4520	−0.0432	0.046*	
C9	0.8368 (3)	0.55503 (13)	0.04948 (16)	0.0365 (5)	
C10	0.8507 (3)	0.61312 (14)	−0.03424 (16)	0.0405 (5)	
C11	0.9361 (3)	0.69144 (14)	−0.01112 (19)	0.0463 (6)	
H11	0.9448	0.7304	−0.0672	0.056*	
C12	1.0081 (3)	0.71168 (15)	0.09455 (18)	0.0468 (6)	
H12	1.0627	0.7651	0.1097	0.056*	
C13	1.0003 (3)	0.65342 (14)	0.17882 (17)	0.0405 (5)	
C14	0.9151 (3)	0.57596 (14)	0.15574 (17)	0.0380 (5)	
H14	0.9094	0.5366	0.2119	0.046*	
C15	1.1064 (3)	0.61377 (16)	0.36363 (18)	0.0576 (7)	
H15A	0.9970	0.5938	0.3785	0.086*	
H15B	1.1726	0.6377	0.4290	0.086*	
H15C	1.1680	0.5658	0.3390	0.086*	
C16	0.8212 (4)	0.63418 (17)	−0.22690 (18)	0.0649 (8)	
H16A	0.7839	0.6936	−0.2260	0.097*	
H16B	0.7679	0.6070	−0.2938	0.097*	
H16C	0.9445	0.6324	−0.2216	0.097*	

### Atomic displacement parameters (Å^2^)

**Table:**

	*U*^11^	*U*^22^	*U*^33^	*U*^12^	*U*^13^	*U*^23^
O1	0.0799 (12)	0.0477 (11)	0.0280 (8)	−0.0122 (9)	0.0054 (8)	0.0022 (7)
O2	0.0909 (13)	0.0381 (10)	0.0523 (10)	−0.0194 (9)	−0.0042 (10)	0.0057 (8)
O3	0.0727 (11)	0.0384 (9)	0.0312 (8)	−0.0072 (8)	0.0064 (7)	−0.0044 (7)
O4	0.0792 (12)	0.0540 (11)	0.0359 (9)	−0.0129 (9)	0.0045 (8)	0.0054 (7)
O5	0.0636 (11)	0.0464 (10)	0.0445 (9)	−0.0064 (8)	−0.0026 (8)	−0.0036 (8)
N1	0.0569 (12)	0.0289 (10)	0.0321 (10)	−0.0078 (9)	0.0098 (8)	−0.0038 (8)
N2	0.0502 (11)	0.0294 (10)	0.0384 (10)	−0.0049 (8)	0.0101 (9)	−0.0028 (8)
C1	0.0414 (12)	0.0287 (12)	0.0293 (11)	0.0003 (9)	0.0075 (9)	0.0018 (9)
C2	0.0394 (12)	0.0395 (13)	0.0283 (11)	0.0013 (10)	0.0082 (9)	0.0015 (10)
C3	0.0469 (13)	0.0365 (13)	0.0343 (12)	−0.0012 (10)	0.0078 (10)	0.0097 (10)
C4	0.0487 (14)	0.0327 (13)	0.0440 (13)	−0.0046 (10)	0.0058 (11)	0.0039 (10)
C5	0.0797 (18)	0.0412 (14)	0.0332 (13)	−0.0130 (13)	−0.0014 (12)	0.0002 (11)
C6	0.0657 (16)	0.0386 (13)	0.0300 (12)	−0.0073 (11)	0.0052 (11)	0.0064 (10)
C7	0.0451 (13)	0.0342 (12)	0.0315 (12)	0.0049 (10)	0.0080 (10)	0.0016 (10)
C8	0.0487 (14)	0.0341 (13)	0.0333 (12)	−0.0013 (10)	0.0076 (10)	−0.0035 (10)
C9	0.0415 (13)	0.0312 (12)	0.0378 (12)	0.0006 (10)	0.0095 (10)	0.0001 (10)
C10	0.0467 (14)	0.0383 (13)	0.0365 (12)	0.0003 (10)	0.0071 (11)	0.0011 (10)
C11	0.0558 (15)	0.0357 (13)	0.0478 (14)	−0.0033 (11)	0.0099 (12)	0.0075 (10)
C12	0.0521 (15)	0.0331 (13)	0.0550 (15)	−0.0036 (10)	0.0090 (12)	0.0000 (11)
C13	0.0420 (13)	0.0370 (13)	0.0421 (13)	−0.0014 (10)	0.0061 (10)	−0.0039 (10)
C14	0.0429 (13)	0.0329 (12)	0.0393 (12)	0.0012 (10)	0.0098 (10)	0.0036 (9)
C15	0.0654 (17)	0.0599 (17)	0.0439 (14)	−0.0057 (13)	−0.0006 (13)	0.0041 (12)
C16	0.089 (2)	0.0674 (19)	0.0395 (14)	0.0021 (16)	0.0143 (14)	0.0098 (13)

### Geometric parameters (Å, °)

**Table:**

O1—C2	1.355 (2)	C5—C6	1.364 (3)
O1—H1	0.8200	C5—H5	0.9300
O2—C4	1.351 (2)	C6—H6	0.9300
O2—H2	0.8200	C8—C9	1.455 (3)
O3—C7	1.251 (2)	C8—H8	0.9300
O4—C10	1.367 (2)	C9—C10	1.389 (3)
O4—C16	1.424 (3)	C9—C14	1.396 (3)
O5—C13	1.375 (2)	C10—C11	1.384 (3)
O5—C15	1.421 (3)	C11—C12	1.374 (3)
N1—C7	1.348 (2)	C11—H11	0.9300
N1—N2	1.376 (2)	C12—C13	1.387 (3)
N1—H1A	0.8600	C12—H12	0.9300
N2—C8	1.275 (2)	C13—C14	1.371 (3)
C1—C6	1.396 (3)	C14—H14	0.9300
C1—C2	1.403 (3)	C15—H15A	0.9600
C1—C7	1.461 (3)	C15—H15B	0.9600
C2—C3	1.377 (3)	C15—H15C	0.9600
C3—C4	1.376 (3)	C16—H16A	0.9600
C3—H3	0.9300	C16—H16B	0.9600
C4—C5	1.387 (3)	C16—H16C	0.9600
			
C2—O1—H1	109.5	C9—C8—H8	120.7
C4—O2—H2	109.5	C10—C9—C14	118.8 (2)
C10—O4—C16	117.56 (18)	C10—C9—C8	121.21 (19)
C13—O5—C15	117.08 (17)	C14—C9—C8	119.95 (19)
C7—N1—N2	117.31 (17)	O4—C10—C11	123.68 (19)
C7—N1—H1A	121.3	O4—C10—C9	116.29 (19)
N2—N1—H1A	121.3	C11—C10—C9	120.0 (2)
C8—N2—N1	117.29 (17)	C12—C11—C10	120.1 (2)
C6—C1—C2	116.88 (19)	C12—C11—H11	119.9
C6—C1—C7	124.33 (18)	C10—C11—H11	119.9
C2—C1—C7	118.77 (18)	C11—C12—C13	120.8 (2)
O1—C2—C3	117.07 (18)	C11—C12—H12	119.6
O1—C2—C1	121.30 (19)	C13—C12—H12	119.6
C3—C2—C1	121.64 (19)	C14—C13—O5	124.6 (2)
C4—C3—C2	119.61 (19)	C14—C13—C12	119.0 (2)
C4—C3—H3	120.2	O5—C13—C12	116.4 (2)
C2—C3—H3	120.2	C13—C14—C9	121.2 (2)
O2—C4—C3	122.76 (19)	C13—C14—H14	119.4
O2—C4—C5	117.22 (19)	C9—C14—H14	119.4
C3—C4—C5	120.0 (2)	O5—C15—H15A	109.5
C6—C5—C4	120.1 (2)	O5—C15—H15B	109.5
C6—C5—H5	120.0	H15A—C15—H15B	109.5
C4—C5—H5	120.0	O5—C15—H15C	109.5
C5—C6—C1	121.75 (19)	H15A—C15—H15C	109.5
C5—C6—H6	119.1	H15B—C15—H15C	109.5
C1—C6—H6	119.1	O4—C16—H16A	109.5
O3—C7—N1	119.56 (19)	O4—C16—H16B	109.5
O3—C7—C1	120.54 (18)	H16A—C16—H16B	109.5
N1—C7—C1	119.89 (18)	O4—C16—H16C	109.5
N2—C8—C9	118.67 (19)	H16A—C16—H16C	109.5
N2—C8—H8	120.7	H16B—C16—H16C	109.5
			
C7—N1—N2—C8	176.80 (19)	N1—N2—C8—C9	−178.34 (17)
C6—C1—C2—O1	−176.99 (19)	N2—C8—C9—C10	−166.4 (2)
C7—C1—C2—O1	4.5 (3)	N2—C8—C9—C14	14.8 (3)
C6—C1—C2—C3	2.6 (3)	C16—O4—C10—C11	17.6 (3)
C7—C1—C2—C3	−175.98 (19)	C16—O4—C10—C9	−163.9 (2)
O1—C2—C3—C4	176.54 (19)	C14—C9—C10—O4	179.36 (18)
C1—C2—C3—C4	−3.0 (3)	C8—C9—C10—O4	0.6 (3)
C2—C3—C4—O2	−176.9 (2)	C14—C9—C10—C11	−2.1 (3)
C2—C3—C4—C5	1.7 (3)	C8—C9—C10—C11	179.09 (19)
O2—C4—C5—C6	178.7 (2)	O4—C10—C11—C12	178.8 (2)
C3—C4—C5—C6	0.0 (4)	C9—C10—C11—C12	0.4 (3)
C4—C5—C6—C1	−0.4 (4)	C10—C11—C12—C13	1.7 (3)
C2—C1—C6—C5	−0.8 (3)	C15—O5—C13—C14	14.2 (3)
C7—C1—C6—C5	177.6 (2)	C15—O5—C13—C12	−166.4 (2)
N2—N1—C7—O3	0.6 (3)	C11—C12—C13—C14	−1.9 (3)
N2—N1—C7—C1	−178.22 (17)	C11—C12—C13—O5	178.70 (19)
C6—C1—C7—O3	170.6 (2)	O5—C13—C14—C9	179.43 (19)
C2—C1—C7—O3	−11.0 (3)	C12—C13—C14—C9	0.0 (3)
C6—C1—C7—N1	−10.6 (3)	C10—C9—C14—C13	1.9 (3)
C2—C1—C7—N1	167.86 (19)	C8—C9—C14—C13	−179.3 (2)

### Hydrogen-bond geometry (Å, °)

**Table:**

*D*—H···*A*	*D*—H	H···*A*	*D*···*A*	*D*—H···*A*
O1—H1···O3	0.82	1.76	2.495 (2)	148
O2—H2···O3^i^	0.82	1.92	2.664 (2)	151
N1—H1A···O1^ii^	0.86	2.17	3.012 (2)	166

Symmetry codes: (i) −*x*+1, *y*−1/2, −*z*+1/2; (ii) *x*, −*y*+1/2, *z*−1/2.
